# Cerebral vasculitis related to neurosarcoidosis: a case series and systematic literature review

**DOI:** 10.1007/s00415-024-12868-2

**Published:** 2025-01-15

**Authors:** Jan K. Focke, Mosche Brokbals, Jana Becker, Roland Veltkamp, Diederik van de Beek, Matthijs C. Brouwer, Willeke F. Westendorp, Markus Kraemer

**Affiliations:** 1 Department of Neurology, Alfried Krupp Hospital, Essen, Germany; 2 Department of Psychiatry and Psychotherapy, LVR Hospital, Düsseldorf, Germany; 3 Department of Psychiatry and Psychotherapy, Florence Nightingale Hospital, Düsseldorf, Germany; 4 Department of Brain Sciences, Imperial College London, London, UK; 5 Department of Neurology, Amsterdam University Medical Centre, Amsterdam, The Netherlands; 6 Department of Neurology, Heinrich Heine University Hospital, Düsseldorf, Germany

**Keywords:** Neurosarcoidosis, Sarcoidosis, Vasculitis, Stroke, Cerebral ischemia, Intracranial hemorrhage

## Abstract

Cerebral vasculitis is a rare but severe manifestation of neurosarcoidosis (NS) that has received little attention. The aim of the present study was to characterize clinical and diagnostic features as well as potential treatment strategies of cerebral vasculitis related to NS. We assessed 29 patients with cerebral vasculitis related to NS (15 female, mean age at time of diagnosis 45 years, SD = 11.85) among these were four new cases from our hospital records and 25 previously published cases from a systematic literature review. The demographic, clinical, and diagnostic features of those 29 patients with cerebral vasculitis related to NS were compared with a group of 73 NS patients without vasculitic involvement (37 female, mean age at time of diagnosis 47 years, SD = 14.79). Neurologic deficits and MRI abnormalities were significantly more frequent in cerebral vasculitis related to NS than in NS without vasculitic involvement. Patients with cerebral vasculitis related to NS significantly more often presented with headache, motor symptoms, and cognitive and/or behavioral changes. Non-neurologic manifestations of sarcoidosis did not significantly differ in character or frequency between both groups. Glucocorticoids in combination with methotrexate, cyclophosphamide, or infliximab were the most frequently used treatment strategies in cerebral vasculitis related to NS. Within the complex diagnostic work-up that is required in cerebral vasculitis related to NS sufficient angiographic imaging as digital subtraction angiography or MRI vessel wall imaging and tissue biopsy are of particular significance as they can detect vascular changes caused by inflammatory processes.

**Supplementary Information:**

The online version contains supplementary material available at 10.1007/s00415-024-12868-2.

## Introduction

Sarcoidosis is a multisystem inflammatory disorder characterized by noncaseating granulomas. Although the exact pathogenesis remains unknown, a genetic susceptibility in combination with the exposure to an environmental trigger leads to an excessive immune response and hereby causes the disease [1–3]. A broad range of infectious, organic, and inorganic antigens have been discussed as putative trigger factors [2]. Sarcoidosis most frequently affects lungs (90%), skin (15%), eyes (10–30%), liver (20–30%) and lymph nodes (10–20%) [1]. Clinical involvement of the nervous system (neurosarcoidosis) is reported in about 5% of the cases and is associated with increased mortality [1, 4, 5]. However, CNS involvement has been observed much more frequently in postmortem studies, suggesting that neurosarcoidosis (NS) is often asymptomatic or remains unrecognized [6]. The diagnosis of NS is often complicated by the heterogeneous disease presentation and the lack of specific diagnostic tests [7, 8]. Frequently described neurologic manifestations include cranial neuropathy, aseptic meningitis, spinal inflammation and peripheral neuropathy [5, 7, 9]. In contrast to systemic sarcoidosis, a histopathologic confirmation of granulomatous inflammation is often difficult in NS. Imaging studies lack specificity but can help to provide evidence for CNS inflammation and exclude differential diagnoses [8, 10, 11]. Contrast-enhanced magnetic resonance imaging (MRI) of the brain and spinal cord is the most appropriate modality in the diagnostic work-up and later follow-up of NS [8, 12]. Common imaging manifestations of NS are white matter lesions, meningeal involvement (e.g. meningeal thickening or contrast-enhancement), hydrocephalus and spinal involvement [10, 13, 14]. Similar to imaging studies, cerebrospinal fluid (CSF) findings are not specific for NS but can be useful to confirm CNS inflammation and exclude differential diagnoses [8]. Common CSF findings in NS include pleocytosis, elevated protein, elevated IgG index, and presence of oligoclonal bands [5, 9, 14]. Commonly used diagnostic criteria for NS based on the above-mentioned clinical and diagnostic characteristics have been proposed by Zaijcek et al. and modified by Marangoni et al. (Table 1) [11, 15].

**Table 1:** Modified Zajicek criteria for the diagnosis of neurosarcoidosis

Definitive	Clinical presentation suggestive of neurosarcoidosis with exclusion of differential diagnoses and histologic confirmation of nervous system involvement
Probable	Clinical presentation suggestive of neurosarcoidosis with evidence of CNS inflammation (elevated levels of CSF protein and/or cells, the presence of oligoclonal bands and/or MRI evidence compatible with neurosarcoidosis) and exclusion of differential diagnoses together with evidence for systemic sarcoidosis (positive histology and/or at least two indirect indicators as fluorodeoxyglucose positron emission tomography (FDG-PET), gallium scan, high-resolution chest computed tomography, bronchoalveolar lavage (BAL))
Possible	Clinical presentation suggestive of neurosarcoidosis with exclusion of differential diagnoses where the above-mentioned criteria are not met

In a population-based study, the hazard ratio for cerebrovascular events in a cohort of 345 patients with sarcoidosis compared to an age- and sex-matched cohort without sarcoidosis was 10.06 during the first 5 years after diagnosis and 1.87 during the following 5 years [16]. The authors of the study put forward several possible explanations for the observed increased risk for vascular diseases—among those the atherogenic effect of chronic inflammatory processes as well as prothrombotic effects of glucocorticoids which were commonly used in the cohort with sarcoidosis [16]. However, another cause of cerebrovascular events in patients with sarcoidosis is cerebral vasculitis associated with sarcoidosis. Although clinical reports on cerebral vasculitis related to NS are limited to case reports or small case series, granulomas inside or around the vascular wall are a frequent finding in histopathological studies with small-sized perforating arteries being preferentially affected [17–19]. Consistently, a recent imaging study found vascular involvement in 9 of 13 NS patients who underwent high-resolution MRI vessel wall imaging (VWI) [20]. Due to the lack of data about clinical and diagnostic characteristics of cerebral vasculitis related to NS, its diagnostic work-up and treatment remains a challenge for practitioners.

The present study aimed to achieve a better understanding of cerebral vasculitis related to NS by characterizing its clinical and diagnostic features in comparison to NS without vasculitic involvement. Another goal was to describe therapeutic strategies for cerebral vasculitis related to NS.

## Methods

### Retrospective case series

We searched the digital hospital information system (SAP Industry Solution Healthcare) of Alfried Krupp Hospital Essen, Germany (AKH) for patients treated in the Department for Neurology from January 2010 to April 2023 with International Statistical Classification of Diseases and Related Health Problems (ICD-10) codes related to Sarcoidosis (i.e., D86.0, D86.1, D86.2, D86.3, D86,8, D86.9, G83.2). 83 patients were identified whose medical records were then screened for the diagnosis of neurosarcoidosis (NS) based on the modified Zajicek criteria [11, 15]. From the 26 patients we identified through this second step, one was excluded from further analysis because of the coexisting diagnosis of primary progressive multiple sclerosis as possible distractor. Among the 25 remaining patients diagnosed with NS we found four who were also diagnosed with a cerebral vasculitis.

### Systematic literature review

**Fig. 1:**
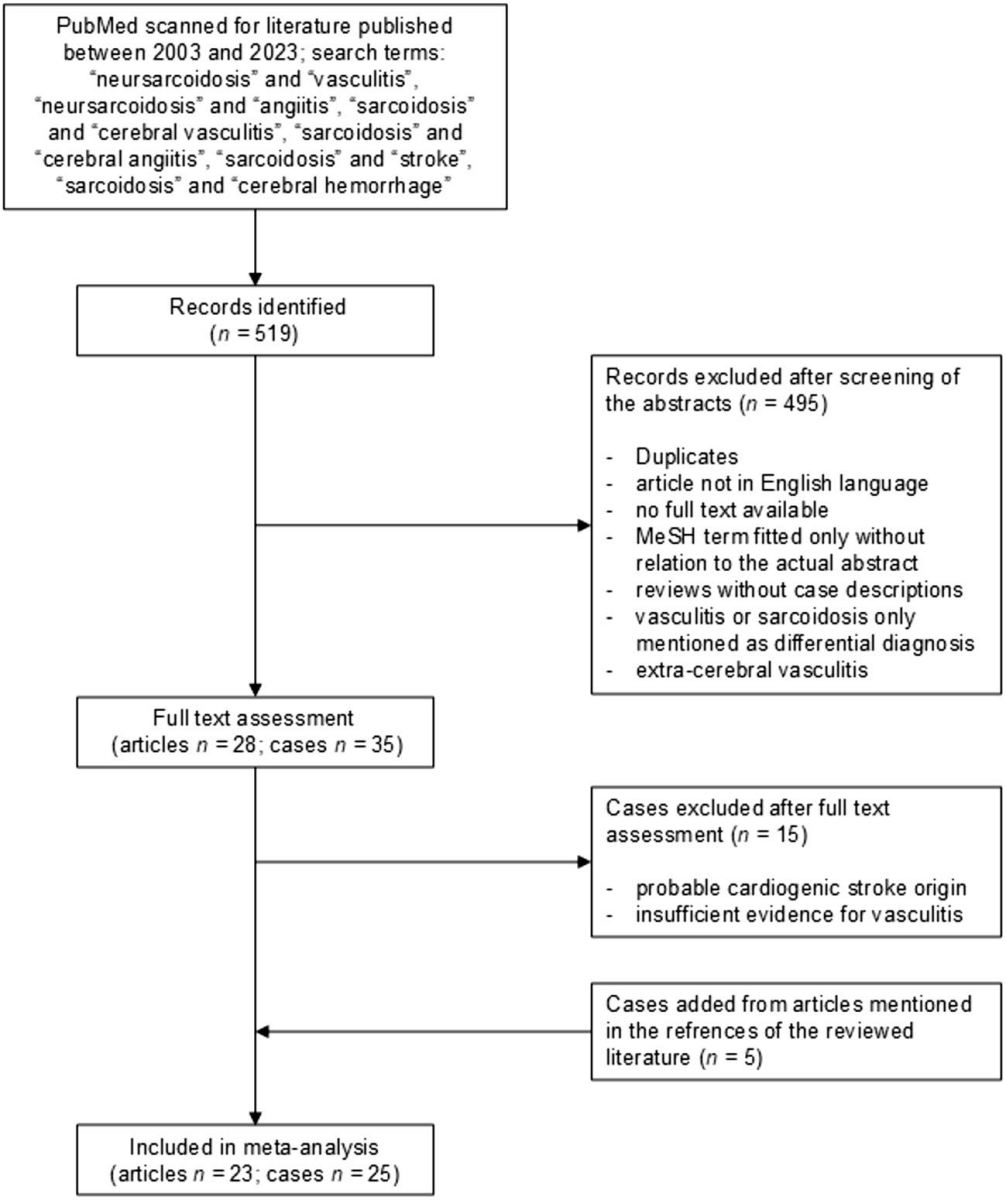
Flow chart of the literature review process

Figure 1 illustrates the working processes of the literature review as a flow chart. To identify additional cases of cerebral vasculitis related to NS PubMed was scanned for literature published in English language between 2003 and 2023 using the search terms “neurosarcoidosis” and “vasculitis”, “neurosarcoidosis” and “angiitis”, “sarcoidosis” and “cerebral vasculitis”, “sarcoidosis” and “cerebral angiitis”, “sarcoidosis” and “stroke”, “sarcoidosis” and “cerebral hemorrhage”. In total, 519 records were found. All abstracts were individually screened for cases of cerebral vasculitis related to NS. Most results (495) were excluded due to following reasons: duplicates, article not in English language, MeSH term fit only without relation to the actual abstract, review without case descriptions, vasculitis or sarcoidosis only mentioned as differential diagnosis, extra-cerebral vasculitis. The remaining 28 articles with 35 individual cases underwent a full text assessment in which 11 cases were excluded because of a probable cardiogenic stroke origin without signs of cerebral vasculitis as competing source of stroke, and four cases were excluded because of insufficient diagnostic evidence of cerebral vasculitis. Three new articles with five case descriptions which were mentioned in the analyzed literature were added. Eventually, 23 articles with 25 cases were included in the meta-analysis [21–43]. To create comparability the cases from the literature were categorized by us in accordance with the modified Zajicek criteria [11, 15]. The level of diagnostic evidence for cerebral vasculitis in the group of case from the literature was heterogeneous. Within the 25 case reports included in this study, three groups can be differentiated according to the level of diagnostic reliability for the presence of cerebral vasculitis: (1) cases of a cerebrovascular event with histopathologic evidence of cerebral vasculitis, and no evidence for a competing cause (3 cases) [21, 26, 41]. (2) cases of a cerebrovascular event with angiographic evidence of vasculitis or with contrast enhancement of cerebral vessel walls on MRI, and no evidence for a competing cause (12 cases) [22–25, 29, 30, 32, 36, 37, 40, 42, 43]. (3) cases of a cerebrovascular event without angiographic or histopathologic evidence of vasculitis, but no better explanation for the cerebrovascular event (10 cases) [27, 28, 31, 33–35, 38, 39, 41].

### Control group

To improve statistical power a cohort of 52 NS patients that were previously reported by the Academic Medical Centre in Amsterdam (AMC) was added to our control group of NS patients without cerebral vasculitis [9]. The data for the present study was partially collected from the preexisting data set of the original publication by Leonhard et al. Parameters that had not been collected as part of the original publication were added from AMC database [9]. Similar to other cohorts, the diagnosis of NS was categorized in accordance with the modified Zajicek criteria [11, 15].

### Data collection and analysis

The demographic and medical characteristics of the patients were collected from the medical records or case descriptions. For the meta-analysis, we pooled the data of the patients from AKH (*n* = 4) and the patients from the literature review (*n* = 25) with cerebral vasculitis related to NS (group 1) and compared this group with the pooled cohort of NS patients without vasculitic involvement from AKH (*n* = 21) and AMC (*n* = 52) (group 2). Due to the retrospective character of our study and the heterogeneity of case descriptions in the literature, not all characteristics could be assessed in every patient. Consequently, categorical variables are presented as the number of patients in whom the respective characteristic was present in relation to the total number of patients in whom it was described (n/N (%)). For continuous variables, the data are presented as mean with standard deviation (SD). For comparison of the two groups (NS with vs. without cerebral vasculitis) chi-square tests were conducted using GraphPad^®^ Prism 6 (GraphPad Software Inc., San Diego, USA) for categorical variables. For continuous data, Kruskal–Wallis-H tests were performed for comparison of the two groups using PSPP Version 1.6 (Free Software Foundation Inc., Boston, USA). A p-value below 0.05 was considered significant.

## Results

Clinical, laboratory and radiographic characteristics as well as biopsy results are summarized in Table 2, results of cerebrospinal fluid analysis in Table 3. Non-neurologic manifestations of sarcoidosis were similar between groups (attachment 1). The different treatment strategies are summarized in Table 4.

**Table 2:** Patient characteristics

	Group 1			Group 2			*p* value
	*n*	*N*	%	*n*	*N*	%	
Isolated NS (a)	9	29	31	18	73	25	0.510
NS in systemic sarcoidosis (a)	20	29	69	55	73	75	0.510
Probability of NS							
Definitive (a)	8	29	28	3	73	4	
Probable (a)	16	29	55	44	73	60	
Possible (a)	5	29	17	26	73	36	
Clinical manifestation of NS							
First manifestation with cerebrovascular event (a)	19	29	66	3	73	4	** < 0.001**
≥ 1 abnormality suggesting NS (a)	29	29	100	64	73	88	**0.048**
Cranial neuropathy (a)	12	29	41	21	73	29	0.219
Headache (a)	20	29	69	23	73	32	** < 0.001**
Sensory symptoms (a)	11	29	38	36	73	49	0.298
Motor symptoms (a)	14	29	48	15	73	21	**0.005**
Ataxia/gait disturbance (a)	8	29	28	14	73	19	0.352
Cognitive/ behavioral changes (a)	8	29	28	6	73	8	**0.010**
Epileptic seizure (a)	7	29	24	3	21	14	0.390
PNS manifestations (a)	0	29	0	16	73	22	**0.006**
Others (a)	19	29	66	6	21	29	**0.010**
Laboratory findings							
Serum ACE increased (b)	3	17	18	23	58	40	0.094
Serum lysozyme increased (b)	0	5	0	23	47	49	nA
Serum neopterin increased (b)	0	4	0	6	19	32	nA
Serum sIL-2R increased (b)	2	4	50	9	21	43	nA
Serum calcium increased (b)	0	2	0	3	44	7	nA
CSF findings							
Lumbar puncture performed (a)	23	29	79	51	73	70	0.335
CNS inflammation (c)	18	23	78	46	51	90	0.165
Pleocytosis (> 5 leucocytes/ml) (b)	18	22	82	37	51	73	0.399
Elevated total protein (> 400 mg/l) (b)	14	19	74	28	51	55	0.154
Elevated IgG-Index (b)	5	9	56	11	41	27	0.094
CSF-specific oligoclonal bands (b)	3	12	25	11	38	29	0.791
CSF lysozyme increased (b)	3	4	75	9	19	47	nA
CSF neopterin increased (b)	4	4	100	17	19	89	nA
CSF sIL-2R (b)	0	4	0	4	19	21	nA
Imaging							
MRI (a)	29	29	100	58	73	79	**0.008**
≥ 1 abnormality suggesting NS (d)	29	29	100	40	58	69	** < 0.001**
Leptomeningeal abnormalities (e)	15	29	52	16	40	40	0.334
Parenchymal T2-hyperintensities (e)	19	29	66	22	40	55	0.380
Parenchymal enhancement (e)	9	29	31	14	40	35	0.730
Abnormalities suggesting stroke (e)	17	29	59	3	40	8	** < 0.001**
Intracranial hemorrhage (e)	9	29	31	1	40	3	** < 0.001**
Spinal lesions (f)	3	5	60	17	31	55	0.829
Others (e)	5	29	17	1	13	8	0.414
DSA/ MRA	20	29	69	6	73	8	**0.0001**
≥ 1 abnormality suggesting vasculitis (g)	12	20	60	0	6	0	**0.010**
DSA abnormalities suggesting vasculitis (g)	10	20	50	nA	nA	nA	nA
Caliber irregularities (h)	7	12	58	0	0	0	nA
Stenosis (h)	8	12	67	0	0	0	nA
Aneurysms (h)	3	12	25	0	0	0	nA
Biopsy confirming sarcoidosis	25	29	86	45	73	62	**0.016**
Cerebral (i)	8	25	32	2	45	4	**0.002**
Pulmonary (i)	10	25	40	36	45	80	** < 0.001**
Others (i)	11	25	44	10	45	22	0.057

a *N* = all patients; b *N* = patients with record of the regarding parameter; c *N* = all patients who recieved lumbare puncture; d *N* = patients who received MRI; e *N* = patients with MRI abnormalities suggesting NS; f *N* = patients who received spinal MRI; g *N* = patients who received vascular imaging; h *N* = patients DSA/MRA abnormalities suggesting vasculitis; i *N* = patients with biopsy confirming sarcoidosis; *sIL-2R* soluble Interleukin 2 Receptor, *CSF* cerebrospinal fluid, *MRI* magnetic resonance imaging, *DSA* digital subtraction angiography, *MRA* magnetic resonance angiography,values in bold are statistically significant

**Table 3:** Results of cerebrospinal fluid analysis

	Group 1				Group 2				*p* value
	Mean	SD	Range	*N*	Mean	SD	Range	*N*	
CSF findings									
CSF white cell count (leucocytes/µl)	103.00	142.87	11–540	13	52.17	66.15	6–268	18	0.173
CSF total protein (mg/l)	1.219	967	470–3710	11	865.06	475.83	407–1931	16	0.199
CSF lysozyme (ng/ml)	4.25	0.35	3.9–4.6	2	3.07	1.80	1.5–6.9	7	–
CSF neopterin (ng/ml)	5.20	1.78	2.5–6.9	4	4.66	4.10	2.1–15	14	–
CSF sIL-2R (U/ml)	nA	nA	nA	0	135.43	35.69	129–182	3	–
Laboratory findings									
Serum ACE (U/l)	151.00	74.00	77–225	2	79.53	11.28	70.2–98.6	4	–
Serum lysozyme (mg/l)	nA	nA	nA	0	30.05	16.13	18.2–73.9	10	–
Serum neopterin (ng/ml)	nA	nA	nA	0	3.94	1.70	2.8–7.3	5	–
Serum sIL-2R (U/ml)	975.50	186.50	789–1162	2	1512.29	537.57	818–2433	7	–
Serum calcium	nA	nA	nA	0	nA	nA	nA	0	–

*N* = patients with elevation of the regarding parameter and exact value available; Upper limit of normal: CSF cell count 5/µl, CSF total protein > 400 mg/l, CSF lysozyme > 62 ng/ml, CSF neopterin > 1.5 ng/ml, CSF sIL-2R > 50 U/ml, serum ACE > 70 U/l, serum lysozyme > 17 mg/l, serum neopterin > 2,5 ng/ml, serum sIL-2R > 623 U/ml

**Table 4:** Immunosuppressive treatment

Treatment	Group 1		Group 2		*p* value
	*n*	%	*n*	%	
Glucocorticoid	27	100	64	96	0.264
Infliximab	6	22	16	24	0.864
Rituximab	1	4	1	1	0.502
Azathioprine	1	4	17	25	**0.016**
Cyclophosphamide	6	22	1	1	** < 0.001**
Methotrexate	9	33	22	33	0.963
Others	5	19	4	6	0.061

*N* = all patients with record of treatment (27 patients in group; 67 patients in group 2), values in bold are statistically significant

### Group 1: patients with neurosarcoidosis and cerebral vasculitis

#### Demographics

**Fig. 2:**
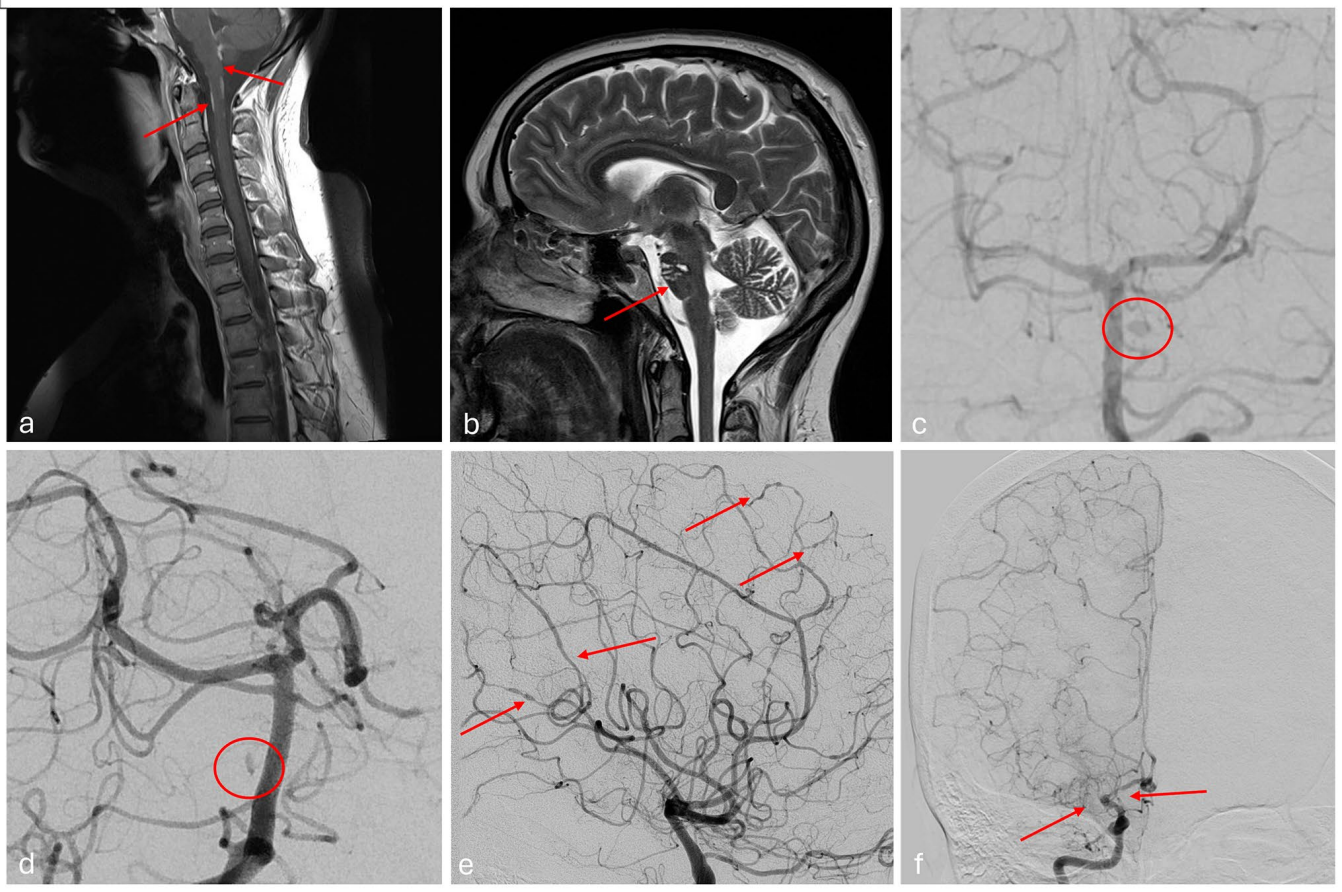
Neuroradiological characteristics of 4 patients with cerebral vasculitis related to neurosarcoidosis **a** MRI with leptomeningeal gadolinium enhancement in patient 1 from Table 5. **b** MRI with gliosis of brainstem in patient 1 from Table 5. **c** Angiogram with one microaneurysm of a pontine perforator artery in patient 1 from Table 5. **d** Angiogram with one microaneurysm of a pontine perforator artery in patient 4 from Table 5. **e** Angiogram with bilateral multiple caliber irregularities in patient 2 from Table 5. **f** Angiogram with moyamoya-like vasculitis in patient 3 from Table 5

In total, 29 patients were included (4 from the records of AKH (Table 5, Fig. 2) and 25 from the literature). Slightly more than half of the patients were female (*n* = 15/29; 52%) with a mean age of 45 years (SD = 11.85; range 21–75).

**Table 5:** Cases of cerebral vasculitis in neurosarcoidosis from our hospital records included in this study

Age/Sex	Neurologic manifestation	Imaging	Diagnostic work-up	Follow-up and Immunosuppressive treatment
No.1 36 yo female	Sensory symptoms Gait disturbance	*2017* CT: acute infarcts of the brainstem and the right occipital lobe DSA: multiple microaneurysms of the pontine perforators *2018* MRI: new infarcts of the brainstem and the right occipital lobe, leptomeningeal contrast-enhancement cerebral as well spinal *2019* MRI: infarct of the left occipital lobe, new leptomeningeal contrast-enhancement	CSF: lymphocytic pleocytosis (68 /µl), protein ↑, OCB typ IV, CSF lysozyme and neopterin ↑ Antineural antibodies neg., laboratory screening for systemic vasculitis neg Event recorder since 07/2017: no atrial fibrillation HRCT of the thorax: Biliary and pulmonary lymphadenopathy Bronchoscopy + BAL + lymph node biopsy 2018 confirmed sarcoidosis CVRF: obesity, hypercholesterolemia	*2017* + *2018* + *2019* Recurring ischemic strokes *2018* → high-dose IV GC, followed by oral GC tapering + RTX + MTX for further GC tapering *2019* Persisting CSF pleocytosis in follow-up lumbar puncture and new leptomeningeal contrast-enhancement in MRI despite treatment with oral GC + RTX → High-dose IV GC + IFX *2020* Improving CSF und MRI results under RTX, IFX and low-dose oral GC 2021 Persisting CSF pleocytosis, steady MRI results → MTX reintroduced *2022* CSF unremarkable, steady MRI + DSA results
No. 2 44 yo male	Uveitis anterior Left-sided hemiparesthesia Motor impairment of the left hand	*01/2023* MRI: acute right-sided thalamic infarct *02/2023* DSA: bilateral caliber irregularities of MCA, ACA and PCA *02/2020* Spinal MRI: unremarkable	CSF lymphocytic pleocytosis (142/ul), protein ↑, OCB typ IV, CSF lysozyme and neopterin ↑ Antineural antibodies neg., laboratory screening for systemic vasculitis neg HRCT of the thorax and abdomen: no sign of systemic sarcoidosis CVRF: smoking	*01/2023* Uveitis anterior *01/2023* thalamic stroke → Oral GC tapering + IFX *02/2023* Improving CSF pleocytosis (32/µl) *08/2023* Frontoparietal brain edema in MRI, progressive angiographic abnormalities and increasing CSF pleocytosis (67/µl) despite oral GC + IFX → RTX *04/2024* MRI regression of the brain edema, CSF unremarkable
No. 3 60 yo female	Transitory left-sided hemianopsia Gait disturbance Headache Cognitive impairments	*01/2020* MRI: leptomeningeal contrast-enhancement, lacunary post-ischemic abnormalities of the right corona radiata *01/2020* DSA: Occlusion of proximal right MCA and left PCA, caliber irregularities of the left MCA, moyamoya-like *01/2020* Spinal MRI: unremarkable	CSF lymphocytic/ monocytic pleocytosis (14/µl), protein slightly ↑, OCB typ IV, CSF lysozyme and neopterin ↑, Antineural antibodies neg., laboratory screening for systemic vasculitis neg., dsDNA-antibodies slightly ↑, Lupus anticoagulant and anti-cardiolipin-antibodies neg., Serum sIL-2R ↑ HRCT of the thorax: Billiary and pulmonary lymphadenopathy Hepatic tissue biopsy 2011 confirmed sarcoidosis CVRF: hypercholesterolemia	*01/2020* cognitive impairments, headache and gait disturbance → High-dose IV GC, followed by oral GC tapering *02/2020* TIA of the right PCA → IFX + oral GC → improving CSF and steady MRI results 06/2022 focal epileptic seizures → anti-epileptic medication
No. 4 48 yo female	Vertigo Headache Left-sided hemihypesthesia Dysarthria Paraparesis Bladder dysfunction	*10/2021* MRI: acute lacunary stroke of the Medulla Oblongata, old postischemic lesions of brain stem and semioval center *11/2021* spinal MRI: spinal lesion Th 10–12 with contrast-enhancement DSA: microaneurysm of the pontine perforators	CSF lymphocytic/ monocytic pleocytosis (11/µl), protein slightly ↑, OCB typ IV, CSF neopterin ↑, Antineural antibodies neg., MOG- AQP4-antibodies neg., Serum sIL-2R ↑ HRCT of the thorax + ultrasound of the abdomen: no sign of systemic sarcoidosis CVRF: smoking	*05/2021* TIA of the brain stem *10/2021* ischemic stroke of the medulla oblongata *11/2021* transverse myelitis Th 10–12 → High-dose IV GC + 4 courses of plasmapheresis followed by oral GC tapering + IFX

*ACA* anterior cerebral artery, *BAL* bronchoalveolar lavage, *CSF* cerebrospinal fluid, *CT* computed tomography, *CVRF* cardiovascular risk factors, *CYC* Cyclophosphamide, *DSA* digital subtraction angiography, *GC* glucocorticoids, *HRCT* high-resolution computed tomography, *IFX* Infliximab, *IV* intravenous, *MCA* middle cerebral artery, *MRI* magnetic resonance imaging, *MTX* Methotrexate, *OCB* oligoclonal bands, *PCA* posterior cerebral artery, *RTX* Rituximab, *sIL-2R* soluble interleukin-2 receptor, *TIA* transitory ischemic attack

#### Systemic features

In accordance with the modified Zajicek criteria (Table 1), a definitive NS was diagnosed in 8 of 29 cases (28%), a probable NS in 16 of 29 (55%), and a possible NS in 5 of 29 (17%). In 20 of 29 cases, a non-neurologic manifestation of sarcoidosis was present (69%). Biopsy confirmed sarcoidosis in 25 of 29 cases (86%). In eight cases, histologic evidence of CNS involvement was obtained. Frequently observed non-neurologic manifestations were lungs or mediastinal lymph nodes (*n* = 20/29; 69%), extra-thoracic lymph nodes (*n* = 11/29; 38%), skin (*n* = 5/29; 17%), and liver or spleen (*n* = 3/29; 10%).

#### Neurological manifestations

All patients (*n* = 29/29; 100%) had at least one neurologic abnormality suggestive of NS. More than half of the patients (*n* = 19/29; 66%) had a cerebrovascular event (ischemic stroke or intracerebral hemorrhage (ICH)) as first clinical manifestation of NS. Patients with cerebral vasculitis related to NS suffered a cerebrovascular event at a mean age of 45 years (SD 11.58; range 26–73). Common neurologic manifestations were headache (*n* = 20/29; 69%), motor symptoms (*n* = 14/29; 48%), cranial neuropathy (*n* = 12/29; 41%), sensory symptoms (*n* = 11/29; 38%), cognitive or behavioral changes (*n* = 8/29; 28%) and ataxia or gait disturbance (*n* = 8/29; 28%).

#### CSF and serum studies

A lumbar puncture was performed in 23 patients (*n* = 23/29; 79%) and revealed signs of CNS inflammation (CSF pleocytosis, elevated protein, elevated IgG-Index, CSF specific oligoclonal bands) in 18 of those patients (*n* = 18/23; 78%). A pleocytosis was present in 18 of 23 patients (78%), hence in all patients with CSF signs of CNS inflammation. Among patients with pleocytosis the exact cell count was reported in 13 patients, the average cell count in these patients was 103 leucocytes/µl (SD = 142.87, range 11–540). CSF total protein level was elevated in 14 of 19 patients (74%). Among patients with elevated total protein the exact value was reported in 11 patients with an average level of 1219 mg/l (SD = 967.31; range 470–3710). IgG-Index was elevated in 5 of 9 (56%) and CSF specific oligoclonal bands were found in 3 of 12 (25%). CSF neopterin was elevated in four of four patients (100%) with an average value of 5.2 ng/ml (SD = 1.78; range 2.5–6.9) and CSF lysozyme was elevated in 3 of 4 (75%) with an average value of 4.25 mg/l (SD = 0.35; range 3.9–4.6), whereas serum neopterin and lysozyme were not elevated in these patients. CSF sIL-2R was not found elevated in the patients with records of this parameter (*n* = 0/4), serum sIL-2R was elevated in 2 of 4 patients (50%).

#### Imaging studies

A cranial and/or MRI was available in all patients (*n* = 29/29; 100%) and revealed abnormalities in all patients (*n* = 29/29; 100%). Leptomeningeal thickening or contrast-enhancement were present in 15 of 29 (52%), parenchymal T2-hyperintensities were shown in 19 of 29 (66%), parenchymal contrast-enhancement was present in 9 of 29 (31%), diffusion-restriction or other abnormalities suggesting ischemic stroke were present in 17 of 29 (59%), and intracerebral hemorrhage in 9 of 29 (31%). Spinal lesions were found in 3 of 5 patients (60%). Magnet resonance angiography (MRA) or catheter angiography were performed in 20 of 29 cases (69%) and revealed abnormalities suggestive of vasculitis in 12 of 20 patients (60%). %). In 10 cases, the abnormalities were revealed by catheter angiography. In 10 cases, the abnormalities were revealed by catheter angiography.

#### Therapy

Immunosuppressive treatment of NS was recorded in 27 of 29 patients (93%). All patients with a record of treatment received glucocorticoids (*n* = 27/27; 100%). In 16 of 27 cases (59%), another treatment in addition to glucocorticoids was recorded. Methotrexate was administered in 9 of 27 cases (33%), infliximab in 6 of 27 (22%) and cyclophosphamide in 6 of 27 (22%). Rituximab and azathioprine were only administered in one case each (*n* = 1/27; 4%).

### Group 2: patients with neurosarcoidosis

In total, 73 patients were included (21 from the records of AKH and 52 from AMC). Slightly more than half of the patients were female (*n* = 37/73; 51%) with a mean age of 47 years (SD = 14.79; range 13–81). Clinical and diagnostic features are summarized in Tables 3–5.

### Group comparison

Among all NS patients from AKH included in this study 4 of 25 (16%) were diagnosed with cerebral vasculitis in NS. The age of the patients did not significantly differ between the two groups. The prevalence of NS in systemic sarcoidosis was higher than of isolated NS in both groups and did not differ significantly between groups (69% vs. 75%; *p* = 0.510). Cerebral vasculitis related to NS was found more often in patients with a systemic sarcoidosis than in isolated NS. Clinical abnormalities suggestive of NS were found significantly more often in group 1 (100% vs. 88%; *p* = 0.048). A cerebrovascular event was significantly more often the clinical first manifestation of NS in group 1 than in group 2 (66% vs. 4%; *p* < 0.001). Significant differences in neurologic manifestations were found between groups in the incidence of headache (69% vs. 32%; *p* < 0.001), motor symptoms (48% vs. 21%; *p* = 0.005), cognitive/ behavioral changes (28% vs. 8%; *p* = 0.010), PNS involvement (0% vs. 22%; *p* = 0.006) and others (66% vs. 29%; *p* = 0.010). Both groups did not significantly differ in the frequency of inflammatory CSF findings. CSF cell count and total protein level of patients were higher in group 1, although the comparisons missed statistical significance (cell count 103 leucocytes/µl vs. 52 leucocytes/µl; *p* = 0.173; total protein 1219 mg/l vs. 865 mg/l; *p* = 0.199). Neopterin, lysozyme and sIL-2R were not compared between groups since these parameters were only reported in four cases of group 1. The incidence of MRI abnormalities suggestive of NS was significantly higher in group 1 (100% vs. 69%; *p* < 0.001). The incidence of leptomeningeal involvement, parenchymal T2-hyperintensities, spinal involvement, and other radiologic abnormalities did not differ significantly between the groups. MRI abnormalities suggestive of ischemic stroke (59% vs. 8%; *p* < 0.001) and ICH (31% vs. 3%; *p* < 0.001) were found significantly more often group 1. Regarding the immunosuppressive treatment groups did not significantly differ in the usage of glucocorticoids, infliximab, rituximab, and methotrexate. Patients from group 1 were significantly more often treated with cyclophosphamide (22% vs. 1%; *p* < 0.001), whereas a treatment with azathioprine was significantly more often used in group 2 (4% vs. 25%; *p* = 0.016).

## Discussion

The present study amalgamates a large cohort of patients diagnosed with cerebral vasculitis related to NS including four new cases from our institute and 25 cases from the literature. It also systematically assesses and compares clinical and diagnostic features of patients diagnosed with cerebral vasculitis related to NS with NS patients without evidence of cerebral vasculitis. Our data emphasize the clinical relevance of cerebral vasculitis related to NS as it is associated with a significantly higher prevalence of MRI abnormalities and neurologic deficits than NS without vasculitic involvement. Furthermore, the study shows that headache, motor symptoms and cognitive changes are more frequent in cerebral vasculitis related to NS. Knowledge of the condition’s symptomatology can help to identify suspected cases of cerebral vasculitis related to NS and direct them to a more intensive diagnostic work-up.

The prevalence of cerebral vasculitis within the cohort of patients diagnosed with NS at our institute (AKH) between 2010 and 2023 was 16% (*n* = 4/25). Although this figure may not be representative for the general population due to a potential selection bias as the AKH is a level one stroke center and member of the European Reference Network on Rare Multisystemic Vascular Diseases, it aligns with other recent studies suggesting that the prevalence of vasculitic manifestations that previously reported [10, 30]. Consistent with other recent reports, cerebral vasculitis related to NS was found more often in patients with a systemic sarcoidosis than in isolated NS [44]. However, this is probably due to the generally higher prevalence of NS in systemic sarcoidosis than isolated NS, since group 1 and 2 did not significantly differ regarding the prevalence of systemic sarcoidosis.

Cerebrovascular events often serve as the first manifestation of NS in patients with vasculitis, occurring at a younger age than in the general population. Intriguingly, several of the patients with a cerebrovascular event as first manifestation of NS had MRI abnormalities at the time of diagnosis suggesting earlier clinically covert strokes. Cerebral vasculitis related to NS was associated with a higher rate of neurologic deficits and MRI abnormalities than NS without vasculitic involvement. These findings highlight the significant clinical impact of cerebrovascular inflammation in NS, likely due to the destructive nature of vasculitis-related events. This is consistent with previous studies that link cerebrovascular events in sarcoidosis to increased mortality [17].

In our series headache, motor symptoms and cognitive and/or behavioral changes were significantly more often present in patients with cerebral vasculitis related to NS. Headache is a common symptom of cerebral vasculitis. Motor symptoms are more frequent in cerebral vasculitis related to NS probably due to the large neural destruction resulting from cerebrovascular events with a higher likelihood of impairing the motor cortex or pyramidal fibers. Progressive cognitive and/or behavioral changes are also a common clinical feature of cerebral vasculitis. Brown and coworkers reported that NS patients with evidence of cerebral vasculitis at autopsy more often presented with encephalopathy than with stroke [19].

These findings suggest that a juvenile cerebrovascular event in sarcoidosis should prompt a focused diagnostic work-up for cerebral vasculitis and—if NS is not already diagnosed—for granulomatous inflammation of the CNS. Also, headache, motor symptoms and cognitive and/or behavioral changes should raise suspicion for cerebral vasculitis, although those symptoms are unspecific and may be difficult to recognize as signs of vasculitis. Because of the increasing number of studies showing a higher prevalence of cerebrovascular events in NS than traditionally thought some researchers even suggested a diagnostic evaluation for vascular involvement in all NS patients [20]. However, due to its rarity there are no validated diagnostic guidelines for cerebral vasculitis related to NS. Moreover, the diagnostic work-up of cerebral vasculitis in general constitutes a clinical challenge as it demands resource intensive and invasive ancillary investigations. According to the European Stroke Organisation guidelines on primary angiitis of the CNS digital subtraction angiography (DSA) and brain biopsy are the most reliable tools in the diagnostic process [45]. Although not specific for vasculitis, CSF studies and MRI of the brain can be helpful in differential diagnostic considerations and are therefore widely applied in clinical practice.

Inflammatory CSF abnormalities—e.g. pleocytosis, elevated protein levels and sometimes oligoclonal banding—are a typical but unspecific finding in patients with cerebral vasculitis and are also frequently encountered in NS [5, 9, 46, 47]. In our study signs of CNS inflammation—as defined above—were found in 78% of the CSF samples of patients with cerebral vasculitis related to NS and their prevalence did not significantly differ between NS patients with and without vasculitic involvement. The pleocytosis in patients with vasculitis related to NS was about twice as high as in NS patients without vasculitic involvement. The CSF total protein levels were also higher in patients with cerebral vasculitis related to NS. Although—due to the lack of statistical power—the comparison between groups missed significance for both parameters, these findings indicate a higher inflammatory burden in patients with cerebral vasculitis related to NS. Various CSF biomarkers have been examined in patients with NS—e.g., soluble Interleukin 2 Receptor (sIL-2R), neopterin and lysozyme—however, the diagnostic value of these parameters remains unclear [9, 48–50]. Our findings indicate good sensitivity of CSF neopterin for NS as it was elevated in 91% of all patients with NS and record of this parameter (4 of 4 in group 1 and 17 of 19 in group 2). CSF lysozyme and sIL-2R do not seem to be of diagnostic value according to our data (see Table 3). Due to the small number of patients with record of the CSF biomarkers in group 1 (*n* = 4) no conclusions about their diagnostic value in cerebral vasculitis related to NS can be drawn from this study. Furthermore, all mentioned CSF biomarkers are lacking specificity as they are also elevated in other neurologic conditions—e.g. neuro-Behçet’s disease (neopterin), neurotuberculosis, CNS lymphoma (sIL-2R), and bacterial meningitis (lysozyme) [48–50].

Conventional MRI of the brain shows a high sensitivity for CNS vasculitis [46, 47, 51]. In our study, patients with cerebral vasculitis related to NS significantly more often presented MRI abnormalities than NS patients without vasculitic involvement. However, MRI abnormalities associated with CNS vasculitis are not specific as they have a broad range of differential diagnoses, among those NS itself. For example, tumor-like changes are found on MRI in isolated CNS vasculitis, in infections and, of course, in neoplastic processes [52, 53]. Imaging of brain vessels plays a central role in the diagnostic work-up of cerebral vasculitis. In clinical practice several techniques are in use to detect vascular abnormalities associated with vasculitis. However, it should be borne in mind that imaging techniques largely differ regarding their diagnostic accuracy. DSA remains the gold standard among vascular imaging techniques in the diagnostic work-up of cerebral vasculitis as—due its high spatial and temporal resolution—it is the only technique that can visualize medium-sized vessels of the intracranial circulation (LIT) [45–47, 51]. In addition, microaneurysms cannot be assessed reliably with the MRI field strength usually applied in clinical practice (except 7 Tesla), which is another advantage of DSA [45, 54]. Further, DSA can help to detect competing causes for cerebrovascular events such as non-inflammatory vasculopathies [46]. Abnormalities that were repeatedly found in DSA of patients with cerebral vasculitis related to NS were caliber irregularities, microaneurysms and stenoses (Fig. 2). Interestingly, in two patients from our institution DSA revealed microaneurysms of the pontine perforator arteries which—in primary angiitis of the CNS—is an untypical finding (Table 2). Despite its diagnostic power, DSA has its limitations as it lacks specificity, cannot prove inflammation and is not fit to detect small-vessel vasculitis [46, 47, 51]. The latter is an especially significant disadvantage in the evaluation of cerebral vasculitis related to NS as small-sized vessel are most frequently affected in this condition [30]. Although not as sensitive as DSA, magnetic resonance angiography (MRA) can also help to detect signs of vasculitic changes of the cerebral blood vessels and is less invasive than conventional DSA [47]. To date however, the diagnostic accuracy of MRA in cerebral vasculitis—especially in comparison to DSA—is not sufficiently addressed in the literature [45]. High-resolution MRI vessel wall imaging (VWI) is an emerging technique in the diagnostics of CNS vasculitis that has already shown its potential in the evaluation of cerebrovascular changes in NS [20, 47]. Although it offers promising possibilities, such as to provide evidence of inflammatory pathologies of the vessel wall, VWI—just like the other imaging techniques mentioned above—is not yet able to visualize small vessels of the CNS. Moreover, although VWI may be able to differentiate vasculitic changes from arteriosclerosis or moyamoya angiopathy in retrospective series, more data from prospective studies is needed to confirm these results [45, 47, 55]. In this study, VWI was not collected systemically. Consequently, tissue biopsy remains the diagnostic gold standard for cerebral vasculitis, especially if small-sized vessel involvement is suspected [45–47, 51]. Accordingly, our data shows a significantly higher frequency of brain biopsy in cerebral vasculitis related to NS than in NS without vasculitic involvement. Due to the lack of specificity of angiographic findings, a biopsy can be helpful even if DSA yielded abnormalities [47]. However, it should be noted that the sensitivity of brain biopsy is limited and it is crucial to avoid sampling errors. On the other hand, the selection of a brain lesion for biopsy can be complicated by its surgical accessibility [46, 47]. In summary the limited specificity of diagnostic tools makes the diagnostic work-up of cerebral vasculitis related to NS challenging. Nevertheless, the identification of cerebral vasculitis in NS is crucial as vascular involvement is associated with a higher morbidity as our data—in line with previous studies—shows [17].

Glucocorticoids were used as first line treatment in all cases of cerebral vasculitis related to NS analyzed in the present study. This is in line with the general treatment practice in NS as glucocorticoids are considered the most rapidly working therapy [12]. However, in most cases of cerebral vasculitis related to NS assessed in this study a second or third line therapy was established either because the disease was refractory to glucocorticoids or to maintain remission despite glucocorticoids tapering. Regarding this additional therapy the data revealed great heterogeneity, which is probably due to the rarity of the condition and the resulting lack of clinical guidelines. Methotrexate, cyclophosphamide, and infliximab were the most frequently used agents. Interestingly, azathioprine which is a well-established steroid-sparing agent in sarcoidosis and NS was significantly less often used in cases of cerebral vasculitis related to NS than in the cohort of NS without vasculitic involvement [1, 12]. Cyclophosphamide on the other hand has been used significantly more often cerebral vasculitis related to NS, which might be due to its role in the acute treatment of primary CNS vasculitis [46, 47]. Because of the central role of tumor necrosis factor alpha (TNF-α) in the pathophysiology of the granulomatous inflammation, TNF-α antagonists as infliximab have become a promising and increasingly used treatment in sarcoidosis and NS [1, 12, 56]. TNF-α antagonists are also used in the treatment of primary CNS vasculitis [47]. Accordingly, in the present study infliximab was—together with cyclophosphamide—the second commonly used steroid-sparing agent in cerebral vasculitis related to NS. However, it should be borne in mind that TNF-α antagonists can increase disease activity in multiple sclerosis which emphasizes the necessity of a proper diagnostic differentiation of the conditions [57]. Regarding the effectiveness of immunosuppressive treatment, the data from the literature review is sufficient. The four cases of cerebral vasculitis related to NS from our institution suggest that a combination of multiple immunosuppressive drugs is often needed to achieve disease stability (no signs of disease activity in clinical examination, MRI and CSF) (see Table 2). This need for a multi-drug-treatment is rare in autoimmune diseases and illustrates the severity of the inflammatory processes in this condition. However, due to the small number of cases no general conclusions or recommendations can be made concerning favorable treatment regimes.

Our study has several limitations including its retrospective nature and the three different sources of recruitment of patients (our center, AMC cohort and literature research) which may have introduced a heterogeneity in terms of the diagnostic evaluation and interpretation, the moment of follow-up assessments in the course of the disease, and the parameters that were assessed. Moreover, a selection bias may have been introduced as tertiary centers such as AKH and AMC are often taking care of more severely affected patients. However, because NS demands an intensive diagnostic work-up and treatment, most NS patients will be referred to a tertiary care hospital at some point. Third, due to the lack of validated diagnostic criteria for cerebral vasculitis related to NS, the diagnostic work-up and resulting level of diagnostic evidence for cerebral vasculitis in group 1 is heterogeneous. We chose to also include cases in group 1 in which the diagnosis of cerebral vasculitis was not supported by brain biopsy or angiography. Consequently, in these cases the diagnosis has been made mainly based on the exclusion of competing causes for cerebrovascular event together with the presence of a cerebrovascular event and CNS inflammation in CSF analysis. This might weaken the diagnostic differentiation between cerebral vasculitis related to NS and non-inflammatory cerebrovascular event in NS patients and hereby cause a distortion of the results by misdiagnosed cases. However, it lies in the nature of cerebral vasculitis that diagnostic results largely differ depending on the size of the involved vessels. While large and medium-sized vessel vasculitis is typically not recognized by tissue biopsy, small-sized vessel involvement cannot be assessed by angiography [58]. As a result, the diagnosis of cerebral vasculitis always requires a broad diagnostic work-up that partly relies on the exclusion of differential diagnoses. As pointed out by Bathla et al. vasculitis related to NS seems to preferentially involve small vessels, and therefore, might be missed in angiography [30]. Brain biopsy, on the other hand, might yield false negative results due to sampling error and is not always possible because of the associated risks for the patient. Consequently, cerebral vasculitis related to NS is probably underdiagnosed. In view of the rarity of NS, prospective studies which systematically apply both methods—angiography and biopsy—to assess possible vascular processes, are neither feasible nor ethically reasonable.

Despite its limitations, the present study draws a detailed picture of the clinical and diagnostic characteristics of cerebral vasculitis related to NS by systematically assessing the so far largest cohort of patients with this rare condition. Another strength of this study lies in the direct comparison between the cohort of patients with cerebral vasculitis related to NS and a group of NS patients without vasculitic involvement, which helps to reveal unique features and hereby provide guidance to practitioners for the diagnostic work-up of the condition.

## Conclusion

Cerebral vasculitis is a clinically relevant manifestation of NS that might be more frequent than traditionally thought and is associated with a higher prevalence of neurologic deficits and imaging abnormalities. A cerebrovascular event often is the first manifestation of NS with cerebral vasculitis. Cerebral vasculitis related to NS demands for a complex diagnostic work-up including DSA and brain biopsy as the most reliable ancillary investigations. Glucocorticoids in combination with methotrexate, cyclophosphamide, or infliximab are the most commonly used strategies in cerebral vasculitis related to NS.

## Supplementary Information

Below is the link to the electronic supplementary material.

Supplementary file1 (DOCX 15 KB)
