# Supplementary material for: Cerebral vasculitis related to neurosarcoidosis: a case series and systematic literature review
Source: J Neurol. 2025 Jan 15;272(2):135. doi: 10.1007/s00415-024-12868-2 (PMC11735521; doi:10.1007/s00415-024-12868-2)
Supplement: Supplementary file 1 — Supplementary file1 (DOCX 15 KB) [file 415_2024_12868_MOESM1_ESM.docx]

**Attachment 1**

Extra-neurologic manifestations of sarcoidosis

|  | Group 1 | | | Group 2 | | |  |
| --- | --- | --- | --- | --- | --- | --- | --- |
|  | n | N | % | n | N | % | p-value |
| Pulmonary (a) | 20 | 29 | 69 | 47 | 73 | 64 | 0.6602 |
| Eyes (a) | 3 | 29 | 967 | 20 | 73 | 27 | 0.0630 |
| Skin (a) | 5 | 29 | 17 | 11 | 73 | 15 | 0.7855 |
| Liver and/ or spleen (b) | 3 | 29 | 10 | 5 | 73 | 7 | 0.5536 |
| Extra-thoracic lymph nodes (b) | 11 | 29 | 38 | 3 | 21 | 14 | 0.0661 |
| Bones (a) | 1 | 29 | 3 | 2 | 73 | 3 | 0.8485 |
| Heart (a) | 0 | 29 | 0 | 3 | 73 | 4 | 0.2678 |
| Kidney (b) | 2 | 29 | 7 | 0 | 21 | 0 | 0.2193 |
| Pancreas (b) | 1 | 29 | 3 | 0 | 21 | 0 | 0.3900 |
| Others (b) | 3 | 29 | 10 | 1 | 21 | 5 | 0.4726 |

a N = all patients b N = patients with record of the regarding parameter; b N = patients with record of the regarding
